# Lipoprotein alterations in endocrine disorders - a review of the recent developments in the field

**DOI:** 10.3389/fendo.2024.1354098

**Published:** 2024-04-02

**Authors:** Michal Olejarz, Ewelina Szczepanek-Parulska, Marek Ruchala

**Affiliations:** Department of Endocrinology, Metabolism and Internal Medicine, Poznan University of Medical Sciences, Poznan, Poland

**Keywords:** endocrine disorders, lipoproteins, HDL-cholesterol, LDL-cholesterol, triglycerides, lipoprotein (a), apolipoprotein B, apolipoprotein A

## Abstract

Dyslipidemia is one of the most common disorders worldwide, which, if left untreated, results in a multitude of complications. Thus proper diagnostics, which includes identifying of secondary causes of dyslipidemia is crucial. Endocrine disorders are an important cause of secondary dyslipidemia. This paper aims to review the publications on lipoprotein alterations in endocrine disorders from the past two years and provide an overview of the recent discoveries in this dynamically developing and large field. Significant changes in lipoprotein serum concentrations are present in most endocrinological diseases and can be modified with proper treatment. Some lipoproteins have also been proposed as markers in some endocrine diseases, e.g., thyroid carcinoma. From the scope of endocrine disorders, the largest number of studies explored the lipoprotein changes in polycystic ovary syndrome and in women during the menopausal and peri-menopausal period. Even though the association of thyroid disorders with dyslipidemia is already well studied, new research has delivered some exciting findings about lipoprotein alterations in euthyroid patients with either positive antithyroid peroxidase antibodies or reduced sensitivity to thyroid hormones. The problem of the adverse metabolic profile, including dyslipidemia in hypoprolactinemia has been recognized. Moreover, this review describes other significant discoveries encompassing lipoprotein alterations in disorders of the adrenals, thyroid, parathyroid glands, pituitary, and gonads. The up-to-date knowledge of the influence of endocrine disorders and hormonal changes on serum lipoproteins is prudent as it can significantly impact therapeutic decisions.

## 1 Introduction

**Figure 1:**
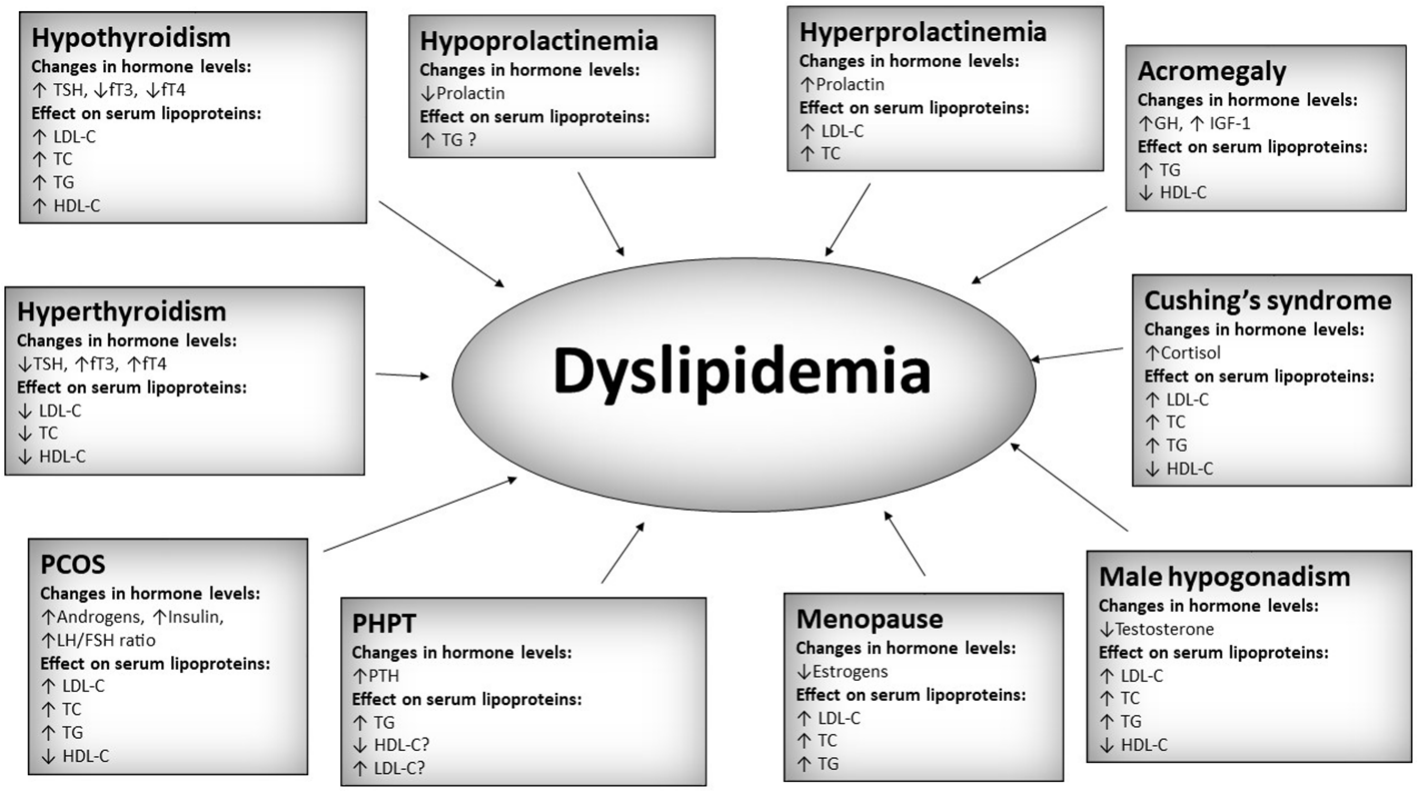
Summary of most important hormonal and lipoprotein changes in selected endocrine disorders. TSH, thyroid stimulating hormone; fT3, free triiodothyronine; fT4, free thyroxine; TC, total cholesterol; LDL-C, low-density cholesterol; HDL-C, high-density cholesterol; TG, triglycerides; PCOS, polycystic ovary syndrome; PHPT, primary hyperparathyroidism; PTH, parathormone; GH, growth hormone; IGF-1, insulin-like growth factor 1. A question mark “?” was added in cases of relatively low amount of data.

Dyslipidemia is defined as an increase in any of the serum lipids (triglycerides, cholesterol, cholesterol esters, phospholipids, lipoproteins) or a decrease in serum high-density lipoprotein (1). It is one of the most common disorders affecting humankind. In 2008, the World Health Organization (WHO) estimated that around 39% of adults worldwide suffer from raised cholesterol levels. The prevalence was the highest in the European Region (54%) and both Americas (48%) (2). Dyslipidemia results in a multitude of complications, especially related to cardiovascular disease: coronary artery disease, peripheral artery disease, ischemic stroke, hypertension, aneurysms, and many more. Cardiovascular-related diseases are the leading cause of death (3). Thus, identifying and treating dyslipidemia is crucial. A standard serum lipid profile usually consists of total cholesterol (TC), low-density cholesterol (LDL-C), high-density cholesterol (HDL-C), and triglycerides (TG). It can be further expanded by the addition of more specialized tests, e.g., intermediate-density lipoproteins (IDL), very low-density lipoproteins (VLDL-C), apolipoprotein A1 (Apo A1), apolipoprotein B (Apo B), lipoprotein (a) [Lp (a)]. Most of the aforementioned particles are proatherogenic and deleterious to human health. However, two of them, namely Apo A1 and HDL-C, are generally considered antiatherogenic. The 2019 European Society of Cardiology emphasizes the importance of Apo B measurement as the Apo B containing lipoproteins have a central causal role in atherosclerosis development (4). The disease is usually caused by a sedentary lifestyle, lack of exercise, wrong diet (excess of calories, high saturated and trans fats intake, alcohol, etc), and genetic factors. However, changes in serum lipoproteins can often be observed in many endocrine disorders. Thus, knowing the associations between different endocrinological diseases and lipid metabolism is prudent. Most commonly, changes in serum lipoproteins occur secondary to the endocrine disorder. However, primary changes in serum lipoproteins may sometimes influence the course of endocrinological diseases. Lipid profile alterations associated with endocrine disorders are generally unfavorable and proatherogenic. Even though in some conditions, e.g., overt hyperthyroidism, a decrease in deleterious lipoproteins like low-density lipoprotein cholesterol (LDL-C) is seen, it cannot be viewed as unequivocally positive since the disease itself is associated with an increased cardiovascular risk (5). The problem of dyslipoproteinemia in endocrinological conditions is immense and would require a very long review. This paper aims to review the recent publications from the last two years on the role of lipoproteins in endocrine disorders and provide an overview of the new discoveries and current research topics in this dynamically developing field in a concise form. The summary of the most important hormonal and lipoprotein changes in selected endocrine disorders mentioned in this review is presented in **Figure 1**.

## 2 Thyroid

### 2.1 Hypothyroidism

Dyslipidemia is a very well-recognized manifestation of overt and subclinical hypothyroidism with thyroid stimulating hormone (TSH) >10 mIU/l. Hypothyroidism can be found in 1.4-13% of patients with hyperlipidemia (6). It is associated with increased TC, LDL-C, Lp (a), and sometimes decreased HDL-C levels (7). However, the data on mild subclinical hypothyroidism with TSH<10 mIU/l are equivocal. A meta-analysis of 35 studies delivered more data on this topic and has shown that even mild subclinical hypothyroidism is associated with an increase in TC, LDL-C, and TG, and a decrease in HDL-C. No differences were reported for VLDL-C, Apolipoprotein B, and Apolipoprotein A serum concentrations (8). Increased anti-thyroid peroxidase antibodies (anti-TPO) are the hallmark of Hashimoto’s thyroiditis diagnosis. Anti-TPO positivity in subclinical hypothyroidism is associated with a higher degree of dyslipidemia. In a cross-sectional study from India, patients with subclinical hypothyroidism and positive anti-TPO had significantly higher TC, LDL-C, and TG levels when compared to antibody-negative subclinical hypothyroid cases (9). Similar relationships were seen in euthyroid subjects with Hashimoto’s thyroiditis, who had higher TC, LDL-C, HDL-C, and TG than healthy euthyroid controls. In this patient group, anti-TPO correlated positively with total cholesterol, LDL-C, and TG (10). However, in another study, the prevalence of anti-TPO in euthyroid subjects was associated only with the presence of low HDL-C without the impact on other lipoprotein serum concentrations (11). The results of the studies mentioned above highlight the need for dyslipidemia screening among patients with anti-TPO positivity. Interestingly, some patients who are euthyroid and don’t have a history of thyroid disease might still be at risk of developing dyslipidemia due to impaired sensitivity to thyroid hormones. A study on 3573 euthyroid participants has shown that reduced thyroid hormone sensitivity is associated with metabolic syndrome and some alterations in lipoproteins, mainly TG (12).

Diagnosing subclinical hypothyroidism using age-adjusted TSH reference values in elderly patients is widely debated. Even though TSH levels are associated with TC, TG, and LDL-C levels in the geriatric population, the relationship varies in different age groups. TSH correlates positively with TC, TG, and LDL-C in the 71-80 age group, with TC in the 65-70 age group, but there was no significant correlation between any lipoproteins and TSH in the >80 years group (13). The impact of TSH on serum lipoproteins seems to be weaker with aging, which further supports the use of age-adjusted TSH reference ranges, at least in the eldest patient groups. However, other conclusions could be drawn from the latest research on Apolipoprotein E (ApoE), a lipoprotein that takes part in various lipid metabolism processes. One of its isoforms, ApoE4, is associated with Alzheimer’s disease. A study by Zhang et al. has found that exosomal transport of liver-derived ApoE4, which causes cognitive decline, is induced by aging-related hypothyroidism and that thyroid hormone levels correlate inversely with the exosomal ApoE4 content. Moreover, in this study, the treatment with levothyroxine has reduced the ApoE4 content of liver and serum exosomes compared with untreated subjects in a mice model (14). The study results are intriguing, but it is yet to be established in further studies if it can prevent cognitive decline.

### 2.2 Hyperthyroidism

Hyperthyroidism is associated with decreased TC, LDL-C, Lp (a), Apo B, and HDL-C serum concentration (7). It is prudent to remember that despite lower levels of the majority of proatherogenic lipid particles, hyperthyroidism is still associated with an increased cardiovascular risk (15). The treatment of overt hyperthyroidism is associated with a reversal of the observed changes – mainly an increase in TC, LDL-C, and HDL-C. However, the impact of subclinical hyperthyroidism treatment on lipid metabolism is uncertain. According to recent studies, definitive subclinical hyperthyroidism therapy is associated foremost with an increase in LDL-C, while the effect on other lipoproteins remains small or uncertain (16, 17). Still, the increase in LDL-C, which is a significant atherosclerosis factor, should make clinicians vigilant of the need for lipid profile check-ups in such patients. Amiodarone-induced hyperthyroidism is one of the most problematic adverse events, which often limits the use of this drug in cardiac patients. Recently, machine learning models, which can predict the occurrence of amiodarone-induced hyperthyroidism, were described. This study found that the most important baseline risk factors for amiodarone-induced thyroid dysfunction were higher HDL-C, TSH, lower free thyroxine(FT4), alkaline phosphatase, and LDL-C, and shorter duration of treatment (18). Such tools are still far from being used in routine clinical practice, but they could significantly improve patients’ safety if available.

### 2.3 Thyroid cancer

Thyroid cancer itself is not associated directly with alternations in serum lipoprotein levels. However, serum lipoprotein levels are routinely evaluated in patients with thyroid cancer after thyroidectomy because of the hormone imbalances that can occur after the surgery. However, recent research suggests they can also be used as a marker for thyroid cancer risk. A study, which contained data from over 3 million participants from health checkups provided by the Korean National Health Insurance Service, has reported that repeatedly measured low HDL-C levels are a risk factor for thyroid cancer. The hazard ratios for thyroid cancer occurrence were 1.243, 1.404, 1.486, and 1.680 for each subsequent low HDL-C measurement. The link of this association was the strongest in patients with metabolic disease, e.g., central obesity, high blood pressure, impaired fasting glucose, and hyperlipidemia (19). The monocyte-to-high-density lipoprotein ratio (MHR) is an inflammation and oxidative stress marker. A recent paper has discovered that patients with papillary thyroid carcinoma (PTC) have higher MHR values. However, the diagnostic accuracy of MHR in diagnosing PTC was borderline acceptable (20). MHR is a very cheap and accessible parameter and could be an interesting adjunctive tool in thyroid nodule workup in the future. However, more research must be performed.

## 3 Primary hyperparathyroidism

Primary hyperparathyroidism is associated with an increased risk of cardiovascular disease. There are some reports about the association of primary hyperparathyroidism with dyslipidemia, mainly consisting of increased TG and, in some populations, also, higher LDL-C and lower HDL-C serum concentrations. However, the data is somewhat inconclusive at this moment (21–24). A recent meta-analysis of 34 studies assessed if parathyroidectomy can modify cardiometabolic risk factors. Unfortunately, no change was found in total cholesterol, LDL, HDL, or triglycerides after surgery. However, the procedure decreased other parameters like glucose, insulin, and blood pressure values (25).

## 4 Polycystic ovary syndrome

Polycystic ovary syndrome (PCOS) is known to be associated with metabolic syndrome and dyslipidemia. All sorts of detrimental lipid profile changes have been reported in PCOS. The lipid profile of PCOS patients is characterized by higher TG, LDL-C, non-HDL-C, and lower HDL-C (26). Recent research on the Chinese population has found that dyslipidemia is present in 41.3% of patients with PCOS and is predominately characterized by low HDL-C. Interestingly, patients with different clinical features of PCOS were found to have different alterations in their lipid profiles. Patients with clinically apparent hyperandrogenism had decreased HDL-C and Apo A serum concentrations. Patients with insulin resistance had increased TG, LDL, Apo B, TG/LDL, and Apo B/Apo A ratios. Patients with dysglycemia or type 2 diabetes had increased TC and TG levels (27). The Apo B/Apo A1 ratio is an interesting parameter, which in PCOS patients is positively associated with the prevalence of metabolic syndrome, its components, aminotransferase elevation, insulin resistance, and correlates with androgen levels (28). Another typical lipoprotein alteration in PCOS is an increased level of lipoprotein (a) compared to healthy controls, which has been recently confirmed by a meta-analysis of 23 studies (29). Dyslipidemia in PCOS is not only associated with increased cardiovascular risk but also with the risk of adverse pregnancy-related outcomes. A recent study by Cai et al. on PCOS women who underwent ovulation stimulation shows that increased total TC, LDL-C, TG, and Apo B are associated with a lower chance of clinical pregnancy and live birth, with total cholesterol, LDL-C, and Apo B is also related to the risk of miscarriage. From the above-mentioned lipid profile parameters, high levels of Apo B seem to be the most important, with odds ratios for pregnancy, live birth, and miscarriage standing at 0.34, 0.39, and 3.82, respectively (30). However, another study that examined 1470 women with PCOS who underwent their first cycle of *in vitro* fertilization/intracytoplasmic sperm injection reported no differences in cumulative pregnancy outcomes among the dyslipidemia and the control groups, with the exception of a negative association between total cholesterol serum concentrations and the cumulative live birth rate (31).

Currently, the standard first-line therapy in PCOS is combined oral contraceptives(COC), with anti-androgen medication being usually the second-line therapy. The efficacy of anti-androgens in PCOS has been evaluated in a large meta-analysis, which has shown that combination therapy with COC and anti-androgens results in a worsened lipid profile with higher TC, LDL-C, and TG (32). The addition of letrozole (an aromatase inhibitor) to ethinylestradiol and cyproterone acetate tablets can result in the improvement of a multitude of hormonal and metabolic parameters when compared to ethinylestradiol and cyproterone acetate therapy alone. The observed lipoprotein changes included lower TC, LDL-C, TG, and higher HDL-C levels. Notably, such treatment was not associated with any differences in the number of adverse events (33).

Natural medicine therapies and nutraceuticals are getting increasing attention in PCOS. In a double-blind, randomized, placebo-controlled trial, the supplementation of crocin resulted in a significant increase in HDL-C and stabilized cholesterol, LDL, and TG levels, which rose during the follow-up period in the placebo group (34). Fenugreek is another option for plan-based treatment for women with PCOS. Two recent studies showed that a Fenugreek seed extract has a beneficial effect on serum lipoproteins, causing a small but significant increase of HDL-C after 12 weeks of therapy in one study (35) and a decrease in TC, LDL-C, and TG when compared to placebo in another trial (36). The benefits of propolis administration in PCOS have been examined by a recent randomized, triple-blinded, placebo-controlled trial. The use of Propolis was associated with reducing the LDL-C to HDL-C ratio (37). A meta-analysis of the three so far published randomized controlled trials about the effects of resveratrol treatment in PCOS did not show any important benefit on serum lipoprotein concentrations apart from lowering total cholesterol levels (38). Even though the changes in serum lipoproteins caused by the use of nutraceuticals are mild or modest, some of those substances seem to be a beneficial and safe add-on to PCOS therapy.

Phthalates are esters of phthalic acid, which are used as plasticizers and belong to the group of endocrine disruptors. They also have an impact on serum lipoprotein levels in women with PCOS. The sum of phthalates in the urine correlates with LDL-C, TG, and TC to HDL-C ratio, while Mono-methyl-phthalate urine levels are associated with TC, LDL-C, TG, and TC to HDL-C ratio (39). Thus, exposure to phthalates should be avoided to minimize the degree of dyslipidemia in PCOS patients.

Idiopathic hirsutism is one of the differential diagnoses of PCOS and, besides PCOS, one of the most common causes of hirsutism in women. In a recent study on 334 patients with idiopathic hirsutism, Mahmoudieh et al. showed that contrary to PCOS, idiopathic hirsutism is not associated with any changes in serum lipoprotein levels or other metabolic risk factors (40).

## 5 Menopause

Estrogens have a crucial role in maintaining correct lipid metabolism. Thus, in the peri-menopausal and post-menopausal periods, when the serum concentrations of estrogens drop, dyslipidemia becomes more prevalent in women. Menopause is associated with higher lipoprotein levels, including higher TG, TC, and LDL-C. However, no difference in HDL-C is observed between pre- and postmenopausal women (41). A study by Karppinen et al. has examined changes in the circulating metabolome by targeted nuclear magnetic resonance metabolomics in women in the transition period from perimenopause to early menopause. In this Finnish cohort study, 85 significant changes in the metabolome were associated with menopause, of which 64 have been directly explained by the hormonal changes during menopause. In this study, the most prominent changes in the metabolome associated with menopause were a significant increase in Apo B, VLDL-C, triglycerides and particles, LDL-cholesterol and particles, and HDL triglycerides, glycerol, and leucine and a decrease in 3-hydroxybutyrate, and citrate serum concentrations. HRT was associated with increased medium-to-large HDL particle count and decreased small-to-medium LDL particle count (42).

A recent meta-analysis of 21 studies involving 1573 women with premature ovarian insufficiency showed that the disease is associated with an adverse lipid profile, consisting of increased total cholesterol, LDL-C, TG, and decreased HDL-C (43). Women who develop premature ovarian insufficiency transit into the postmenopausal period much earlier than healthy women and thus are exposed for a longer time to the proatherogenic changes related to estrogen deficiency. That is why early check-ups of the lipid profile and fast introduction of proper treatment are of utmost importance in those women.

Currently, hormone replacement therapy (HRT) is indicated for the alleviation of vasomotor symptoms of menopause. Research on the influence of HRT on cardiovascular risk is still ongoing, and some very interesting papers on this topic were published recently. A meta-analysis of 14 randomized controlled trials assessed the effect of HRT in the form of 17β-estradiol plus norethisterone acetate on serum lipoprotein (a) and apolipoproteins. The meta-analysis showed that such treatment causes a mean decrease of 67.59 mg/L in lipoprotein (a), of 3.71 mg/dl in Apo B, and has no effect on apolipoprotein A1 and A2 levels (44). A study on nearly 90,000 postmenopausal women recruited from the UK biobank has shown that increased lipoprotein (a) was associated with an increased risk of coronary heart disease. Moreover, it has been found, similarly to the previously described meta-analysis, that HRT decreases lipoprotein (a) levels. However, interestingly, no evidence of lower lipoprotein (a) associated risks was found between HRT users and nonusers (45). Correct timing of HRT and avoiding late HRT commencing might be crucial for preventing harmful changes in HDL-C in postmenopausal women.

## 6 Male hypogonadism

Hypogonadism is associated with an unfavorable lipid profile consisting of increased TC, LDL-C, TG and low HDL-C. However, a recent study on 7268 men participating in a health examination suggests that the association between testosterone and LDL-C is nonlinear. Instead, the study reports an inverted U-shaped association between the prevalence of testosterone <3.5ng/ml and the deciles of LDL-C, thus implying a higher risk of testosterone <3.5 ng/ml in patients with both high and low LDL-C levels. Moreover, a negative association of testosterone with the deciles of TG and a positive with the deciles of HDL-C is also shown (46). The results of this study suggest that not only high LDL-C but also low LDL-C serum concentrations, especially when combined with high TG and low HDL-C, should indicate screening for male hypogonadism.

In recent years, the awareness of testosterone replacement therapy(TRT) and its use in hypogonadal men has increased. There has been a long debate about whether TRT increases the cardiovascular risk in hypogonadal men. Fortunately, a recent multicenter, randomized, double-blind, placebo-controlled trial showed that TRT was non-inferior to placebo in the incidence of major adverse cardiac events (47). Moreover, a meta-analysis of 16 randomized controlled trials showed that TRT significantly lowers LDL-C levels. Still, the effect on HDL remains unclear due to the imprecision or heterogeneity of the analyzed studies (48). Not only testosterone but also gonadotrophins seem to be associated with lipoprotein levels. A recent paper reported that the follicle-stimulating hormone (FSH) in hypogonadal males with type 2 diabetes correlates with TC, LDL-C, and TG levels. Interestingly, this finding was independent of testosterone serum concentration (49). Thus, FSH might be a new marker of dyslipidemia in this patient group.

## 7 Adrenal disorders

It is long known that hypercortisolism is associated with an altered lipid profile. Dyslipidemia can be observed in Cushing’s syndrome of any etiology (pituitary, adrenal, ectopic). Cushing’s syndrome typically presents with increased TC, LDL-C, TG, and decreased HDL-C. Surgical or pharmacological treatment can normalize serum lipoprotein levels, but sometimes dyslipidemia persists even after resolution of hypercortisolemia (50). Less information is available about hyperaldosteronism. A meta-analysis of 30 studies examined the differences in serum lipoproteins between patients with primary hyperaldosteronism and essential hypertension. It has shown that patients with primary hyperaldosteronism have lower triglycerides, LDL-C, and TC levels compared to essential hypertension subjects (51). Even though this observation’s mechanism is unknown, the increased cardiovascular risk in primary aldosteronism patients seems to be unrelated to dyslipidemia.

Interestingly, even in patients with neither clinically apparent signs of adrenal disease nor a presence of an adrenal tumor, the morphology of the adrenal gland might affect its function and impact lipid metabolism. This was shown in a study on 588 diabetic patients without any comorbidities or medications affecting adrenal gland function. Researchers in this study measured adrenal limb thickness and found it to be inversely correlated with HDL-C (52).

## 8 Acromegaly and growth hormone deficiency

Acromegaly is associated with dyslipidemia development, which can occur in up to 74% of patients (53). The main lipoprotein changes observed in acromegaly include hypertriglyceridemia and reduced HDL-C serum concentrations (54). Recent studies have shown, that dyslipidemia improves upon somatostatin analogue treatment. Long-acting somatostatin analog treatment results in a decrease in TG to HDL-C ratio and Lp (a) and an increase in HDL-C. The reduction in mean growth hormone levels is positively associated with the decline in TG, Lp (a), and an increase in HDL-C (55). Pegvisomant is a growth hormone (GH) receptor antagonist that effectively lowers the insulin-like growth factor 1 (IGF-1) levels and improves symptom control in acromegaly. Even though previous studies did not find many alterations in serum lipoproteins after pegvisomant treatment, according to a recent study assessing the outcomes of a 10-year-long, continuous pegvisomant treatment, the use of this drug is associated with a decrease in TC and LDL-C. A combined therapy, consisting of pegvisomant with the addition of a somatostatin analog, has been shown to lower TC and LDL-C even stronger than pegvisomant monotherapy (56).

## 9 Hyperprolactinemia and hypoprolactynemia

Hyperprolactinemia is an established, well-known clinical entity. Lipid profile abnormalities in patients with prolactinomas usually are higher TC and LDL-C serum concentrations (57). However, the potential clinical significance of hypoprolactinemia has been recognized only recently. A recent study reported that women with iatrogenic hypoprolactinemia caused by cabergoline treatment have higher serum concentrations of CRP, fibrinogen, uric acid, HbA1C, 2-h post-challenge glucose, TG, and lower levels of testosterone, free androgen index and HDL-C. Fortunately, all those changes were reversible after cabergoline dose reduction and normalization of prolactin levels (58). The results of this study show that the goal of dopamine agonist treatment should be the achievement of normoprolactinemia, and overtreatment leading to hypoprolactinemia should be avoided.

## 10 Conclusions

The knowledge of lipoprotein alterations in endocrine disorders has developed further. Most of the recent research focused on thyroid disease, menopause, PCOS, and hypogonadism. The up-to-date knowledge of the influence of endocrine disorders and hormonal changes on serum lipoproteins is prudent as it can significantly impact therapeutic decisions.

## Author contributions

MO: Conceptualization, Investigation, Writing – original draft, Writing – review & editing. ES: Conceptualization, Writing – review & editing. MR: Conceptualization, Writing – review & editing.
